# Safety and Growth Optimization of Lactic Acid Bacteria Isolated From Feedlot Cattle for Probiotic Formula Design

**DOI:** 10.3389/fmicb.2018.02220

**Published:** 2018-09-28

**Authors:** Cecilia Aristimuño Ficoseco, Flavia I. Mansilla, Natalia C. Maldonado, Hortencia Miranda, María E. Fátima Nader-Macias, Graciela M. Vignolo

**Affiliations:** Centro de Referencia para Lactobacilos, Consejo Nacional de Investigaciones Científicas y Técnicas, San Miguel de Tucuman, Argentina

**Keywords:** lactic acid bacteria, antibiotic resistance, virulence determinants, growth conditions, feedlot cattle

## Abstract

In order to eliminate the widespread use of antibiotics in livestock production, the research for alternatives has increased lately. This study examined the safety of 40 lactic acid bacteria (LAB) isolated from bovine feedlot environment and previously selected as potential probiotics. A high sensitivity prevalence to ampicillin (AMP, 100%), gentamicin (GEN, 96.3%), kanamycin (KAN, 96.3%), clindamycin (CLI, 85.2%), chloramphenicol (CHL, 92.6%) and streptomycin (STR, 88.9%) while moderate and high resistance against erythromycin (ERY, 48%) and tetracycline (TET, 79%) respectively, were determined. Feedlot enterococci and pediococci displayed high resistance to CLI, ERY, GEN and TET (73, 100, 54.5, and 73%, respectively). Among fifteen resistance genes investigated, seven were identified in lactobacilli; their presence not always was correlated with phenotypic resistance. STR resistance genes, *aad*A and *ant(6)* were observed in 7.4 and 3.7% of isolates, respectively; genes responsible for aminoglycosides resistance, such as *bla* (7.4%), and *aph(3”)-III* (3.7%) were also recognized. In addition, resistance *cat* and *tetS* genes (3.7 and 7.4%, respectively) were harbored by feedlot lactobacilli strains. The presence of *erm*B gene in 22.3% of isolates, including two of the six strains phenotypically resistant to ERY, exhibited the highest prevalence among the assessed antibiotics. None of the feedlot lactobacilli harbored virulence factors genes, while positive PCR amplification for *ace*, *agg*, *fsrA*, and *atpA* genes was found for enterococci. With the objective of producing large cell biomass for probiotic delivery, growth media without peptone but containing glucose and skim milk powder (Mgl and Mlac) were selected as optimal. *Lactobacillus acidophilus* CRL2074, *L. amylovorus* CRL2115, *L. muc*o*sae* CRL2069, and *L. rhamnosus* CRL2084 were strains selected as free of antibiotic resistance and virulence determinants, able to reach high cell numbers in non-expensive culture media and being compatible among them.

## Introduction

In the last 20 years, direct-fed-microbials (DFM) supplementation to improve the health and performance of livestock has generated a great interest. Specifically for feedlot beef cattle, main targets for probiotics are health promotion to avoid or reduce ruminal acidosis, increase weight gain and feed conversion as well as human pathogens shedding decrease (Retta, 2016). Ruminants benefit from the symbiosis between the host and the rumen microbes, which supply protein, vitamins and short chain organic acids for the animal host (Chaucheyras-Durand and Durand, 2010). Defined as “live microorganisms which when administered in adequate amounts confer a health benefit on the host” (FAO, 2002; Hill et al., 2014), probiotic bacteria represent a new and efficient alternative to traditional prophylactic therapies for animal management in artificial environments. The increased interest for DFM application constitute a driving force to reduce or eliminate the use of low-dose antibiotics in livestock production; low antibiotic concentrations found in natural environments lead to enrich resistant bacterial populations (Gullberg et al., 2011). In the European Union, the use of antibiotics for animal growth promotion was banned in 2006 and a similar ban for animal husbandry is currently discussed in United States of America (Cantas et al., 2013). Internationally, many countries have adopted mandatory restrictions on antimicrobial use, and veterinary prescription to use these drugs in food animals are obligatory (Maron et al., 2013).

According to WHO (World Health Organization) to attain a probiotic status, microorganisms have to meet some of the principles related to their safety and biological properties. Lactic acid bacteria (LAB), besides their essential role in food fermentations are also important as probiotics. For this type of use, requirements for safety assessment have increased; they should not exhibit neither pathogenic activity nor antibiotic resistance (AR) encoding genes and sustain genetic stability. The evaluation of the antibiotic susceptibility of LAB has recently grown because of their potential to spread resistance by horizontal gene transfer in which plasmids, transposons and integrons are involved; these mobile elements include AR genes mostly responsible for intra- and inter-species transfer of genetic material (van Reenen and Dicks, 2011; Gueimonde et al., 2013). The large numbers of LAB in fermented products and in the gastrointestinal tract (GIT) supports the presence of different resistance mechanisms via mutation; once a bacteria becomes resistant, the element is amplified and may be transmitted to another host. *Enterococcus* species have been described as a major source of nosocomial infections in human and veterinary medicine and a correlation of AR and infective determinants was established (Garcia-Migura et al., 2014). Enterococci factors that contribute to pathogenesis include cytolysin, aggregation substance, adhesins and hydrolytic enzymes (Franz et al., 2011). Remarkably, food isolated enterococci were shown to harbor either single or multiple virulence factors, however, their incidences among probiotic enterococci strains were noticeably lower (Franz et al., 2011; Beukers et al., 2015; Imperial and Ibana, 2016). Although lower occurrence, AR for non-enterococcal LAB emerged from medical, veterinary and food sources; the presence of potentially transferable resistance genes has been established, which appear to be intrinsic as well as transferable (Ammor et al., 2007; Devirgiliis et al., 2013; Abriouel et al., 2015). Among the microorganisms used in animal feed, mainly Gram-positive bacterial strains that act as bioregulators of the intestinal microbiota and enhancers of host’s natural defenses, were applied (Hill et al., 2014). As normal components of animal GIT microbiota, different genera and species of LAB were used to potentially modulate GI microbial health, nutrient use and animal productivity (Retta, 2016).

On the other hand, as first step for probiotic delivery to feedlot cattle, optimization of an effective and low cost growth medium for culturing LAB must be achieved. Because LAB are fastidious microorganisms and many elements like carbohydrates, amino acids, peptides, vitamins and Mg/Mn salts are required for growth, commercial media are generally optimal, but due to the high cost they result inappropriate for large-scale biomass production. In addition, LAB growth activity is affected by culture conditions such as pH, temperature, medium formulation, and others. Among ingredients, yeast extract was found as highly significant for enhanced biomass production in low cost cultivation conditions (Chiang et al., 2015; Manzoor et al., 2017). In this study, in view to design a probiotic formula for its administration to feedlot cattle, AR and virulence factors incidence for 40 LAB strains isolated from cattle environment, were investigated. In addition, optimal growth conditions of selected probiotic LAB strains were preliminary investigated for high cell mass production.

## Materials and Methods

### Microorganisms and Growth Conditions

Forty LAB previously isolated from feedlot cattle environment and selected for their beneficial characteristics (Maldonado et al., 2018) were used, including *Lactobacillus (L.) acidophilus* (3), *L. amylovorus* (4), *L. casei* (1), *L. fermentum* (1), *L. mucosae* (14), *L. plantarum* (3), *L. rhamnosus* (1), *Pediococcus (P.) acidilactici* (2), *Enterococcus (E.) durans* (3), *E. faecium* (2) and *E. hirae* (6). Inoculum of strains were prepared by transferring glycerol stock culture to MRS broth (Merck, Darmstadt, Germany) and sub-cultured twice in the same media at 37°C for 16 h.

### Safety Evaluation

#### Phenotypic Antibiotic Resistance and MIC Determination

Minimum inhibitory concentrations (MIC, μg/ml) of eight antibiotics: ampicillin (AMP), clindamycin (CLI), chloramphenicol (CHL), erythromycin (ERY), gentamicin (GEN), kanamycin (KAN), tetracycline (TET), and streptomycin (STR) were determined for LAB strains (40) according to ISO 10932:2010 standard. Epidemiological cut-off values based on the recommendation of the European Committee on Antimicrobial Susceptibility Testing (EUCAST) and EFSA-FEEDAP (2012) Panel on Additives and Products or Substance used in Animal Feeding (FEEDAP) were applied. All antibiotics were purchased from Sigma–Aldrich (St. Louis, MO, United States and ICN Biomedicals, Santa Ana, CA, United States). In parallel, accuracy of susceptibility testing was monitored by the use of quality control strains (*Lactobacillus plantarum* ATCC14917, *Enterococcus faecalis* ATCC29212).

#### Hemolysin and Gelatinase Activity

Hemolysin activity was determined on Columbia Blood Agar (Oxoid) containing 5% defibrinized horse blood after 48 h of incubation at 37°C, both under aerobic and anaerobic conditions. Zones of clearing around colonies indicated β-haemolysin production. Gelatinase production was detected by inoculating LAB onto freshly prepared peptone-yeast extract agar containing gelatin (30 g/L; Difco). Plates were incubated overnight at 37°C and cooled at ambient temperature for 2 h. The appearance of a turbid zone around the colonies was considered as positive result for gelatinase production.

#### PCR Detection of Antimicrobial Resistance Genes and Potential Virulence Factors

The presence of genes coding for AR in LAB strains phenotypically susceptible to antibiotics (described above) and virulence factors were evaluated through PCR reactions. Specific primers used and their target genes, amplicon sizes and PCR protocol references used for genes detection are shown in **Table 1**. PCR-amplifications were performed from total bacteria DNA obtained according to Pospiech and Neumann (1995) in 25 μl reaction mixture containing 1 μl of purified DNA, 1 μM of each primers, 0.1 mM of each dNTP (2.5 Mm), buffer 1x, 1.5 mM MgCl_2_ (25 Mm) and 2.5 U/100 μl of Taq polymerase (Inbio Highway, Argentina). Samples were subjected to an initial cycle of denaturation (94°C for 5 min), followed by 28 cycles of denaturation (94°C for 1 min), annealing for 1 min at the temperature of the primer pairs and elongation (72°C for 1 min 30 seg), ending with one cycle of final extension (72°C for 5 min) in a MyCyclerTM (BioRad, Richmond, CA, United States) thermocycler. PCR-products were separated by electrophoresis on 1% (w/v) agarose at 80 V for 45 min. Gels were stained with GelRedTM (Biotium Inc., Hayward, CA, United States) and visualized with a UV light transilluminator (320 nm). The molecular size marker used was 1 kb Plus DNA ladder (Invitrogen, Buenos Aires, Argentina).

**Table 1:** Genes targeting to antibiotic resistance and virulence determinants used in this study.

Primer pair	Target Gene (Antibiotics)	Primer sequence (5′-3′)	Amplicon size (bp)	T°C	References
Bla-F	*bla* (Ampicillin)	CATARTTCCGATAATASMGCC	297	48	Hummel et al., 2007
Bla-R		CGTSTTTAACTAAGTATSGY			
Cat-F	*cat* (Chloramphenicol)	TTAGGTTATTGGGATAAGTTA	300	50	Hummel et al., 2007
Cat-R		GCATGRTAACCATCACAWAC			
erm(B)-F	*erm*(B) (Erythromycin)	CATTTAACGACGAAACTGGC	640	60	Ouoba et al., 2008
erm(B)-R		GGAACATCTGTGGTATGGCG			
erm(C)-F	*erm*(C) (Erythromycin)	CAAACCCGTATTCCACGATT	295	60	Ouoba et al., 2008
erm(C)-F		ATCTTTGAAATCGGCTCAGG			
aac(6′)-aph(2′′)-F	*aac*(6′)*aph*(2′′)	CCAAGAGCAATAAGGGCATA	220	52	Ouoba et al., 2008
aac(6′)-aph(2′′)-R	(Gentamicin)	CACTATCATAACCACTACCG			
aph(3′′)-III-F	*aph*(3′′)-III	GCCGATGTGGATTGCGAAAA	292	60	Ouoba et al., 2008
aph(3′′)-III-R	(Kanamycin)	GCTTGATCCCCAGTAAGTCA			
StrA-F	*str*A (Streptomycin)	CTTGGTGATAACGGCAATTC	548	60	Ouoba et al., 2008
StrA-R		CCAATCGCAGATAGAAGGC			
StrB-F	*str*B (Streptomycin)	ATCGTCAAGGGATTGAAACC	509	57	Ouoba et al., 2008
StrB-R		GGATCGTAGAACATATTGGC			
AadA-F	*aad*A (Streptomycin)	ATCCTTCGGCGCGATTTTG	282	65	Ouoba et al., 2008
AadA-R		GCAGCGCAATGACATTCTTG			
AadE-F	*aad*E (Streptomycin)	ATGGAATTATTCCCACCTGA	565	57	Ouoba et al., 2008
AadE-R		TCAAAACCCCTATTAAAGCC			
ant(6)-F	*ant*(6) (Streptomycin)	ACTGGCTTAATCAATTTGGG	597	60	Clark et al., 1999
ant(6)-R		GCCTTTCCGCCACCTCACCG			
tet(M)-F	*tet*(M) (Tetracycline)	GTGGACAAAGGTACAACGAG	406	57	Ng et al., 2001
tet(M)-R		CGGTAAAGTTCGTCACACAC			
tet(K)-F	*tet*(K) (Tetracycline)	TTAGGTGAAGGGTTAGGTCC	697	57	Aarestrup et al., 2000
tet(K)-R		GCAAACTCATTCCAGAAGCA			
tet(L)-F	*tet*(L) (Tetracycline)	CATTTGGTCTTATTGGATCG	456	57	Aarestrup et al., 2000
tet(L)-R		ATTACACTTCCGATTTCGG			
tet(S)-F	*tet*(S) (Tetracycline)	TGGAACGCCAGAGAGGTATT	660	57	Ouoba et al., 2008
tet(S)-R		ACATAGACAAGCCGTTGACC			
**Primer pair**	**Target Gene (Virulence factors)**	**Primer sequence (5′-30′)**	**Amplicon**		**References**
			size (bp)		
Agg-F	*agg*	AAGAAAAAGAAGTAGACCAAC	1553	53	Espeche et al., 2012
Ace-R	(Aggregation protein)	AAACGGCAAGACAAGTAAATA			
Ace-F	*Ace*	CAGGCCAACATCAAGCAACA	125	65	Al-Talib et al., 2015
Ace-R	(Accessory colonization factor)	GCTTGCCTCGCCTTCTACAA			
EspA-F	*esp*A (Enterococcal surface protein)	TTTGGGGCAACTGGAATAGT	407	60	Al-Talib et al., 2015
EspA-R		CCCAGCAAATAGTCCATCAT			
Ebp-F	*ebp*	AATGTGTTAAACCATCAAGGGAAT	372	62	Sillanpää et al., 2013
Ebp-R	(Endocarditis and Biofilm-associated Pilus)	ACTCCTTTTTGAACTTCACCAATC			
CylA-F	*cyl*A (Cytolisin)	ACTCGGGGATTGATAGGC	688	60	Vankerckhoven et al., 2004
CylA-R		GCTGCTAAAGCTGCGCTT			
HyI-F	*hyI* (Hyaluronidase)	ACAGAAGAGCTGCAGGAAATG	276	62	Vankerckhoven et al., 2004
HyI-R		GACTGACGTCCAAGTTTCCAA			
GelE-F	*gel*E (Gelatinase)	CGAAGTTGGAAAAGGAGGC	372	50	Al-Talib et al., 2015
GelE-R		GGTGAAGAAGTTACTCTGA			
SprE-F	*spr*E (Serineprotease)	GGTAAACCAACCAAGTGAATC	300	57	Al-Talib et al., 2015
SprE-R		TTCTTCCGATTGACGCAAAA			
fsr A-F	*fsr*A	TGATGATGATTGATTGATGGAC	744	60	Qin et al., 2000
fsr A-R	(Quorum sensing genes)	ATTACAAGTGGCACACCAGGAC			
fsr B-F	*fsr*B	TGGACAAAGTATTATCTAACCG	729	57	Qin et al., 2000
fsr B-R	(Quorum sensing genes)	CACACCATCACTGACTTTTGC			
fsr C-F	*fsr*C	ATCGTGTGTTAGAAAATAGC	1344	52	Qin et al., 2000
fsr C-R	(Quorum sensing genes)	ACGAATCACAACCACTAAGTC			
AtpA-F	*atp*A	CCAGGTCGTGAAGCTTATCC	110	63	Šeme et al., 2015
AtpA-R	(F0F1-ATP synthase subunit alpha)	GGTAAGGCCGTCATTGAACC			
cfa1-F	*cfa1*	ACGACCTGTTGTTCGACCTG	150	63	Šeme et al., 2015
cfa1-R	(Cyclopropane-fatty acylphospholipidsynthase)	AGGGGGCTATATCCCAAATG			
mleS-F	*mle*S	ACAAGGTCTCAGCGTTCAGC	140	64	Šeme et al., 2015
mleS-R	(Malatedehydrogenase)	GACTGGGATTCCAGCTGATG			
HisD-F	*his*D	TGAACCACTCGGTGACTACG	150	62	Šeme et al., 2015
HisD-R	(Histidinoldehydrogenase)	GGAGCTTCCTTAGCCAAAGC			
groEL-F	*gro*EL	GTTTGATCGCGGCTATCTGA	150	55	Koirala et al., 2015
groEL-R	(Stress response)	CCTTGTTGMACGATTTCTTG			

### Optimization of Growth Medium for Probiotic Strains Production

Based on safety results, *L. acidophilus* CRL2074, *L. amylovorus* CRL2116, *L. mucosae* CRL2069, and *L. rhamnosus* CRL2084 were selected and the impact of different culture media formulated with various ingredients on biomass production was investigated. Five combinations of different components, MRS and MRSc (pH was controlled by adding NaOH 5N at 6, 18, and 24 h) were evaluated, their compositions being shown in **Table 2**. Before the trial, selected LAB strains were inoculated (2%) in 5 ml of each prepared media and sub-cultured twice during 12 h at 37°C. For each medium and LAB strain, viable LAB were quantified after dilutions and plating on MRS agar. Maximum growth rate (μ h^-1^) and growth potential (CFU/mL at 24 h – CFU/mL at 0 h) were determined.

**Table 2:** Culture media used and their composition.

	Media composition (g L^-1^)					
	Mpep	Mgl	Mlac	Mw^§^	Msm	MRS/MRSc
Peptone casein	9	–	–	–	–	–
Beef peptone	–	–	–	–	–	10
Skim milk powder	–	–	10	–	100	–
Yeast extract	3.12	20	10	3.12	10	5
WPC 80	–	–	–	9	–	–
Beef extract	–	–	–	–	–	10
Glucose	6.27	10	–	6.27	–	20
Lactose	–	–	30	–	–	–
Ammonium citrate	–	2	–	–	–	2
Sodium acetate	–	5	–	–	–	5
L-cistein	0.25	–	–	0.25	–	–
KH_2_PO_4_	–	–	5.6	–	–	2
Na_2_HPO_4_	6.27	–	3.6	6.27	–	–
NaCl	2.5	–	–	2.5	–	–
MgSO_4_cdot7H_2_O	–	0.1	0.05	–	–	0.1
MnSO_4_cdotH_2_O	–	0.05	0.038	–	–	0.05
Tween 80	–	1^∗^	–	–	–	1^∗^
pH	6.5	5.9	6.5	6.5	6.2	6.5

^∗^*Tween 80: ml/l; ^§^ WPC 80: soluble protein concentrate from whey*.

### Compatibility of Selected LAB Strains

Beneficial LAB strains previously selected were tested for interactions among them. MRS (15 ml) melted and tempered at 45°C were vigorously mixed with 200 μL of an overnight culture of each LAB and poured into Petri dishes. Wells of 10 mm in diameter were cut in the agar and 30 μL of the cell-free supernatant of each strain was placed into each well. The plates were incubated aerobically overnight at 37°C, and inhibition halos observed.

### Statistics

Agar assays were performed by duplicate and growth curves by triplicate. In the case of AR, media values were compared with cut-off points. The media and SD were calculated for growth curves, results (means OD ± SD) being evaluated by the application of ANOVA to define differences and statistical significances were determined by the Tukey test.

## Results and Discussion

### Antimicrobial Susceptibility Testing

The use of probiotics instead of antibiotic therapy is gaining acceptance worldwide to alleviate antibiotic-mediated complications and enhance livestock health conditions. However, safety concerns have been raised by the use of LAB strains carrying AR genes themselves, as they can potentially transfer them to other commensal and/or pathogenic bacteria through horizontal gene mechanisms (Imperial and Ibana, 2016). Thus, to use as probiotics, safety traits of forty LAB strains previously isolated and identified from feedlot cattle environment (Maldonado et al., 2018), were investigated. The MIC of eight antimicrobial agents for 40 LAB strains involving *Lactobacillus* (27), *Pediococcus* (2), *Enterococcus* (11) strains, was determined. Results showed that the obtained MICs were in the range (μg/ml) of 0.063-16 (CLI); 0.125-64 (CHL); 0.25-16 (ERY); 0.5-64 (GEN); 1-128 (STR) and 0.5-128 (TET); ≤ 0.032 (AMP) and ≤ 1024 (KAN), as shown in **Table 3**. Feedlot lactobacilli were found resistant to the glycopeptide VAN (data not shown), this phenotype being characterized as an intrinsic resistance in LAB (Gueimonde et al., 2013). Similarly, all strains were sensitive toward the β-lactam AMP in coincidence with that reported for probiotics *L. acidophilus*, *L. rhamnosus*, and *L. casei*, commercial starter *L. plantarum* and *L. mucosae* strains (Hummel et al., 2007; Klose et al., 2014); however resistance toward AMP was described for chicken lactobacilli (Dec et al., 2017). Although resistance to aminoglycosides has been often observed for probiotic and starter lactobacilli (Hummel et al., 2007; Nawaz et al., 2011; Abriouel et al., 2015), GEN, KAN, and STR sensitivity was repeatedly described in feedlot lactobacilli (>92%) (**Table 3**). Only *Lactobacillus* CRL2158 was resistant to GEN, *L. plantarum* CRL2103 exhibited resistance to KAN and *L. acidophilus* CRL2074, *L. amylovorus* CRL2065 as well as *L. mucosae* CRL2155 were resistant to STR. In coincidence, low lactobacilli resistance to aminoglycosides was reported for chicken intestinal LAB strains (Dec et al., 2017). In this study, the low MICs found for feedlot *L. mucosae* strains agrees with that described for wild boars fecal strains (Klose et al., 2014). In addition, resistance to STR of *L. acidophilus* from human origin and *L. amylovorus* from broilers were reported (Cauwerts et al., 2006; Klare et al., 2007). On the other hand, high prevalence of KAN resistance was described for *L. acidophilus*, *L. rhamnosus*, and *L. casei* from probiotic products (Temmerman et al., 2003). Resistance/sensitivity of *L. plantarum* to KAN were found to be controversial, strains isolated from probiotic products and fermented foods were reported as susceptible (Temmerman et al., 2003), while resistance was described by Nawaz et al. (2011). Discrepancies might be assigned to differences in the evaluated species, applied methods or strains source.

**Table 3:** Distribution of MICs and antibiotic resistance genes among **lactobacilli** and **pediococci** isolated from feedlot environment.

LAB	Strain (CRL)	Origin		CLI	CHL	ERY	GEN	KAN	STR	TET	Resistance gene(s)
*L. acidophilus*			*Cut-off value*	*1*	*4*	*1*	*16*	*64*	*16*	*4*	
	2061	CF	MIC	0.063	4	0.5	2	32	2	**64**	
	2074	CF		**2**	4	0.25	0.5	8	**64**	**32**	
	2152	CF		0.125	4	0.5	2	8	2	**128**	*erm*B
*L. amylovorus*			*Cut-off value*	*1*		*1*	*16*	*16*	*16*	*4*	
	2044	CF	MIC	**16**	1	0.25	2	8	1	**16**	
	2065	CF		0.4	2	**8**	2	8	**128**	**16**	
	2115	PS		0.4	4	1	2	4	1	**128**	
	2116	PS		0.4	4	0.25	2	16	1	**64**	
*L. casei*			*Cut-off value*	*1*	*4*	*1*	*16*	*64*	*64*	*4*	
	2088	PS	MIC	0.125	4	**4**	16	32	64	0.5	*aph*(3′′)-III, *aadA*
*L. fermentum*			*Cut-off value*	*1*	*4*	*1*	*16*	*64*	*64*	*8*	
	2085	FR	MIC	0.032	4	0.016	0.5	16	4	4	*erm*B, a*nt*(6), *aadA*
*L. mucosae*			*Cut-off value*	*1*	*4*	*1*	*16*	*64*	*32*	*8*	
	2063	PS	MIC	0.125	**64**	**16**	16	64	32	**64**	
	2064	CF		0.032	2	1	4	32	2	**64**	
	2069	CF		0.032	2	1	4	4	2	**32**	
	2070	PS		0.125	4	0.5	2	16	2	**64**	
	2083	CF		0.063	2	0.25	2	8	2	**64**	
	2100	PS		0.063	2	**4**	0.5	16	2	**16**	
	2101	PS		0.063	4	**4**	0.5	8	2	**128**	*erm*B
	2111	CF		0.063	2	0.25	0.5	2	2	**64**	
	2112	CF		0.063	4	0.5	0.5	0	8	**128**	
	2113	CF		**16**	**64**	**16**	0.5	32	2	**16**	*erm*B
	2114	CF		0.063	2	1	0.5	8	2	**128**	*erm*B
	2154	CF		0.063	4	1	0.5	0	2	**128**	*erm*B
	2155	CF		0.125	2	**32**	16	16	**64**	1	*tet*S
	2158	CF		0.063	0.125	**32**	**32**	8	32	**128**	
*L. plantarum*			*Cut-off value*	*2*	*8*	*1*	*16*	*64*	*n.r*	*32*	
	2103	FR	MIC	**4**	8	**4**	2	**512**	16	32	*bla*
	2126	FR		2	8	**2**	1	32	8	32	*cat*
	2142	FR		0.125	8	**4**	1	32	16	**64**	*bla*, *tet*S
*L. rhamnosus*			*Cut-off value*	*1*	*4*	*1*	*16*	*64*	*32*	*8*	
	2084	FR	MIC	0.25	4	**2**	4	32	8	8	
*P. acidilactici*			*Cut-off value*	*1*	*4*	*1*	*16*	*64*	*64*	*4*	
	2043	CF	MIC	0.032	2	**8**	16	64	16	**16**	
	2046	FR		0.032	2	**8**	16	64	16	**16**	

*Cut-off values proposed by the EFSA-FEEDAP (2012) and MIC are expressed in μg mL^-*1*^; *n.r*, not required; number in bold indicate antibiotic resistance. CF, cattle feces; PS, pens soil; FR, feed rations*.

Generally, lactobacilli were sensitive to antibiotics inhibiting protein synthesis, such as CLI, CHL, ERY, and TET (Ammor et al., 2007; Klare et al., 2007). In agreement, high susceptibility (MICs below the cut-off value) to CLI and CHL was described among feedlot lactobacilli involving 85.2 and 92.6% of strains, respectively. Similar results were reported for lactobacilli isolated from chickens and wild boar feces (Klose et al., 2014; Dec et al., 2017), however, high prevalence of lincosamides (CLI) resistance was published for broilers cloacal lactobacilli (Cauwerts et al., 2006). With the exception of *L. acidophilus* and *L. fermentum*, all other lactobacilli (44.5%) showed to be resistant to ERY. These results are in line with those reported for human and animal *L. rhamnosus, L. amylovorus*, probiotic *L. casei* and meat starter *L. plantarum* strains (Cauwerts et al., 2006; Hummel et al., 2007; Gueimonde et al., 2013) while feedlot *L. mucosae* resistance (43%) toward ERY resulted higher to that reported for wild boars intestinal strains (Klose et al., 2014). Moreover, an unexpected high prevalence of TET resistance was observed among feedlot *Lactobacillus* (78% of the strains) with MICs values far beyond the cut-off value (128 μg/ml). *L. acidophilus* CRL2152, *L. amylovorus* CRL2115 and 4 strains of *L. mucosae* exhibited the highest TET resistance level in agreement with those reported for food and animal feces lactobacilli (Klose et al., 2014; Sornplang et al., 2016).

When feedlot pediococci were analyzed, resistance toward ERY and TET, while sensitivity to the other antimicrobials were obtained (**Table 3**). Susceptibility to AMP, CHL, GEN, and STR is in accordance to previous results (Danielsen et al., 2007; Hummel et al., 2007). *P. acidilactici* (two strains) resistances to ERY and TET agree to that described for food and animal strains (Ammor et al., 2007; Danielsen et al., 2007; Hummel et al., 2007) starter strains (Hummel et al., 2007), respectively. Nonetheless, as reported by Danielsen et al. (2007), pediococci are intrinsically resistant to TET in addition to VAN. On the other hand, enterococci as commensal inhabitants of the GIT of warm-blooded animals were dominant in feedlot environment (Maldonado et al., 2018). Since this genus emerged as important human and veterinary pathogen/opportunist, the incidence of antimicrobial resistance and virulence determinants were also investigated. Feedlot enterococci, mostly isolated form cattle feces, showed sensitivity to AMP, CHL, KAN and STR while resistance to CLI, ERY, GEN and TET (73, 100, 54.5, and 73%, respectively) was displayed (**Table 4**). Susceptibility of enterococci to AMP and CHL is in accordance to that previously reported (Anderson et al., 2008), and similar sensitivity to STR for enterococci isolated from feedlot steers was described by Beukers et al. (2015). In agreement, low incidence (<10%) of KAN and CHL resistance was reported for wild game Spanish meat enterococci (Guerrero-Ramos et al., 2016). Resistance to GEN found in 56% of *Enterococcus* agrees with that reported by Iseppi et al. (2015) for pet animal’s enterococci. The unexpected high percentage of feedlot enterococci resistant to CLI (82%), ERY (100%) and TET (73%) are concordant with that reported for dairy/bison cattle and pet feces (Anderson et al., 2008; Jackson et al., 2010; Iseppi et al., 2015; Beukers et al., 2015). Among the recovered enterococci from feedlot steers feces, *E. hirae* was revealed to predominate (Maldonado et al., 2018) and was also described among the highest antibiotic resistant enterococci species (Beukers et al., 2015). In addition, multi-resistance to at least three antimicrobial agents were found for 30% of feedlot strains in which CLI was mostly involved for enterococci strains (**Table 4**). Fifteen lactobacilli isolates (37.5%) were resistant to only one antibiotic, *L. casei*, *L. plantarum*, and *L. rhamnosus* strains showing ERY with MICs 1 ≥ μg/ml, while *L. acidophilus*, *L. amylovorus*, and *L. mucosae* exhibited TET MICs ≥ 4 μg/ml. Similarly, Klose et al. (2014) reported multi-resistance to CHL/KAN/STR/TET for *L. mucosae* strains isolated from wild boars feces. Specifically for enterococci, multidrug resistance patterns found are in agreement to that reported for *E. hirae*, *E. faecium*, and *E. durans* from dairy cows feces, *E. hirae* being resistant up to seven antimicrobials (Jackson et al., 2010). Nevertheless, *L. fermentum* CRL2085 from feedlot cattle was phenotypically sensitive to all assayed antibiotics in this study, in disagreement to that reported for fermented food and animal/human feces strains which were resistant to ERY and TET (Ammor et al., 2007; Nawaz et al., 2011; Sornplang et al., 2016).

**Table 4:** Distribution of MICs and virulence genes among **enterococci** isolated from feedlot.

LAB	Strain (CRL	Origin	CLI	CHL	ERY	GEN	KAN	STR	TET	Virulence genes
			*1^∗^*	*16*	*4*	*32*	*1024*	*128*	*4*	
*E. durans*	2047	CF	**8**^§^	8	**16**	**64**	1024	32	0.5	*ace*, *agg*
	2048	PS	0.25	8	**16**	**64**	512	32	**128**	
	2153	CF	**8**	8	**16**	32	256	32	0.5	*agg*
*E. faecium*	2102	CF	0.25	16	**16**	32	1024	32	**128**	
	2141	PS	**8**	4	**16**	**64**	256	32	0.5	
*E. hirae*	2062	CF	**16**	4	**8**	**64**	64	32	**128**	
	2067	CF	**8**	4	**8**	**64**	256	64	**128**	
	2068	CF	**16**	8	**8**	32	32	16	**16**	*ace*, *fsrA*
	2071	PS	**16**	4	**8**	8	64	32	**128**	
	2072	CF	0.25	4	**8**	**64**	64	64	**128**	
	2089	CF	**8**	4	**8**	32	128	64	**128**	*atp*A

*^∗^Cut-off values [proposed by the EFSA-FEEDAP (2012)]; ^§^ MICs are expressed in μg mL^-*1*^; n.r, not required; number in bold indicates antibiotic resistance. CF, cattle feces; PS, pens soil; FR, feed rations*.

### Identification of Antibiotic Resistant Genes in Feedlot LAB Strains

Given the high prevalence of CLI, ERY, GEN and TET resistances found for enterococci, only lactobacilli sensitive strains were subjected to PCR amplification for the detection of resistance genes. Antibiotic sensitive LAB strains in which resistance genes have been detected are shown in **Table 3**. Seven of the 15 investigated genes were evidenced in feedlot lactobacilli. Although the presence of these genes were not always phenotypically correlated, molecular determinants for 27.5% of lactobacilli strains were found. While none of the feedlot strains was phenotypically resistant to AMP, PCR analysis showed *L. plantarum* CRL2103/CRL214 strains harboring *bla* genes. Similar results were reported for this lactobacilli species from swine and poultry meat, that even phenotypically negative, were found to carry *blaZ* gene (Aquilanti et al., 2007). In contrast, although phenotypically resistant, Hummel et al. (2007) observed a lack of molecular detection of *bla* gene for *L. plantarum* starter strains. In addition, none of the feedlot lactobacilli was found to host GEN resistance gene, although *L. mucosae* CRL2158 was phenotypically resistant. Nevertheless, the presence of the *aac(6′)aph(2′′)* gene encoding for GEN resistance in lactobacilli isolated from chicken, pigs, pet and wild boars feces, was reported (Ammor et al., 2007; Klose et al., 2014; Dec et al., 2017). Although phenotipically sensitive, the *aph(3′′)-III* gene conferring KAN resistance was present in *L. casei* CRL2088 in coincidence to that described for probiotic strain by Ouoba et al. (2008). Conversely, even when *L. plantarum* CRL2103 exhibited high phenotypic resistance (MIC ≥ 512 μg/ml), KAN resistance gene was absent. Likewise, from the genomic DNA of *L. mucosae* CRL2063/CRL2113 with a resistant phenotype to CHL, *cat* gene could not be amplified. Similarly, the occurrence of this gene was reported in *L. plantarum* CRL2126 with MIC ≥ 8 μg/ml (cut-off value) in coincidence with that reported for strains isolated from probiotic products (Temmerman et al., 2003). When STR resistance genes, *aadA* and *ant(6)* were evaluated, their presence in *L. casei* CRL2088 and *L. fermentum* CRL2085 strains were detected, MICs values were ≤ to cut-off value. The *aadA* gene was present in both lactobacilli strains, whereas *ant(6)* gene only occurred in the STR sensitive *L. fermentum* strain (MIC ≥ 4 μg/ml). Although phenotypic sensitivity to all assayed antibiotics, *L. fermentum* CRL2085 exhibited the co-occurrence of both STR resistance genes. Positive PCR for *aadA* gene in *L. casei* CRL2018 obtained in this study agrees with that reported for food and human strains, but none of the phenotypically resistant or sensitive food *L. fermentum* strains were positive for the investigated STR resistance genes (Ouoba et al., 2008). Resistance to aminoglycosides may result from various mechanisms, such as the lack of cytochrome electron transport responsible for antibiotic uptake, changes in cellular permeability and enzymatic antibiotic modification by acetyl-, adenyl-, and phospho-transferases, whose encoding genes are mostly found on plasmids and transposons (Abriouel et al., 2015).

Furthermore, a prevalence of *erm* and *tet* genes among feedlot lactobacilli was found (**Table 3**). ERY resistance genes were identified as *ermB*, while TET resistance genes belonged to the *tet(S)* class. Six of 27 feedlot lactobacilli harbored *ermB* gene; *L. mucosae* CRL2101/CRL2113/CRL2114/CRL2154 phenotypically resistant to ERY (MICs ≥ 1 μg/ml) as well as *L. acidophilus* CRL2152 and *L. fermentum* CRL2085 susceptible to ERY (MICs ≤ 0.5 μg/ml) displayed positive PCR for *ermB* gene. In contrast to these results, *L. mucosae* strains from wild boars feces did not harbor ERY resistant genes (Klose et al., 2014). The detection of *ermB* as a major resistant gene for this class of antibiotic in bovine cattle LAB is consistent with that previously reported for lactobacilli from various sources (Ammor et al., 2007; Hummel et al., 2007; Klare et al., 2007; Anderson et al., 2008; Nawaz et al., 2011). When TET resistance genes was analyzed, despite its high phenotypic prevalence with MICs far beyond the break point, a low occurrence of *tet*S gene was detected. This gene conferring resistance to TET was only present in the phenotypically resistant *L. plantarum* CRL2142, this being in coincidence with that found from food and human strains (Ammor et al., 2007; Zonenschain et al., 2009; Nawaz et al., 2011), whereas *L. mucosae* CRL2155 with a sensitive phenotype (MIC = 1 μg/ml), was PCR positive for *tetS* gene. Similarly, Klose et al. (2014) reported sensitive *L. mucosae* isolates from wild boars as harboring *tetS* gene. Resistance *tet*S and *erm*B genes were identified on both, plasmids and the chromosome for *Lactobacillus* species from different fermented foods (Nawaz et al., 2011; Abriouel et al., 2015). The high level of resistance to ERY and TET in lactobacilli from feedlot environment is in agreement with the use of these antibiotics in veterinary therapy and for growth promotion in domestic and meat animals (Anderson et al., 2008). Of the resistant lactobacilli and pediococci, eleven strains (28%) carried resistance genes, which was higher than that reported for LAB from dairy, pharmaceutical and probiotic products, in which only 12% of strains were PCR positive. From strains carrying resistance genes, only three correlated with phenotypic results (*L. plantarum* CRL2142 and *L. mucosae* CRL2101/2113 for TET and ERY, respectively). As recently reported by Hughes and Andersson (2017), the lack of correlation between phenotype and genotype may be explained by the intrinsic resistance to the tested antibiotics and the resistance emergence through evolutionary events such as mutations or defective expression of the resistance gene due to environmental and genetic modulation of the phenotypic expression of AR.

### Identification of Virulence Factors

Enterococci and lactobacilli are commensal bacteria of the human and bovine GIT, but are also associated with clinical and community-acquired infections in humans (Franz et al., 2011). Genes encoding virulence factors were studied in feedlot LAB strains and results are shown in **Table 4**. When enzymatic activities were examined, neither gelatinase nor β-hemolytic activities were exhibited by the analyzed LAB strains; all enterococci showed α-hemolysis, while lactobacilli (four *L. mucosae* strains) were also α-hemolytic, the remaining feedlot strains (85%) were γ-hemolytic or non-hemolytic (data not shown). In coincidence, none of the enterococci from pet animal’s feces, food and water were β-hemolytic (Abriouel et al., 2008; Iseppi et al., 2015) although gelatinase activity was described for dairy and pet feces enterococci (Lopez et al., 2006; Iseppi et al., 2015). In addition, the absence of β-hemolysis in feedlot enterococci that correlated with the lack of amplification of *cyl*A gene is in line with that reported for environmental enterococci (Pangallo et al., 2008). None of the feedlot lactobacilli harbored virulence factors genes (data not shown); these are generally regarded as safe due to their long history of presence in the normal GIT of humans and animals and safe use in fermented foods. However, lactobacilli have been associated with several cases of infections such as bacteremia, endocarditis but also with localized infections, *L. casei* and *L. rhamnosus* being common causative agents (Lara-Villoslada et al., 2010).

The frequency of genes encoding virulence factors among the feedlot enterococci strains is shown in **Table 4**. Positive PCR amplification for accessory colonization factor (*ace*), aggregation substance (*agg*), quorum sensing (*fsrA*) and ATP synthase subunit alpha (*atpA*) genes were found for *E. durans* and *E. hirae* strains. In agreement with this result, the absence of genes coding for virulence factors in *E. faecium* from sheep feces was reported (Mannu et al., 2003). In contrast, positive PCR for the other genes here evaluated was described for *E. faecium* from food, clinical and pet feces samples (Abriouel et al., 2008; Iseppi et al., 2015). The presence of *ace* gene was detected in feedlot *E. durans* and *E. hirae* strains, while *agg* gene was found in 2 out of 3 *E. durans* strains, *fsrA* and *atp*A genes being also PCR positive for *E. hirae* feedlot strains. On the contrary, a lack of amplification of the virulence genes here assayed was reported for *E. durans* and *E. hirae* from fermented sausages and pet feces (Martin et al., 2005; Fontana et al., 2009; Iseppi et al., 2015). In particular, *fsrA* gene coding for quorum sensing regulatory mechanism was present in feedlot *E. hirae* CRL2068 in coincidence with that recently described for dairy strains by Popović et al. (2018). The lack of amplification of *fsrA* gene in *E. durans* feedlot strains agrees with that reported for this species by Golińska et al. (2013). In addition, *atpA* gene encoding for the alpha subunit of ATP synthase was present in feedlot *E. hirae* CRL2089 in correlation with the use of this virulence determinant as identification probe for poultry *E. hirae* strains (Champagne et al., 2011). Nevertheless, the absence of *gelE* gene in feedlot enterococci, in coincidence with the lack of gelatinase activity, agrees with that reported by Diarra et al. (2010) for *Enterococcus* species isolated from broilers chicken. Although the low incidence of virulence genes among feedlot enterococci, positive PCR genes were related to adhesion, colonization, biofilm formation and energy metabolism which may facilitate gene transfer in the GIT of meat animals and problematic pathogen lineages might arise.

### Optimization of Growth Conditions for Selected Feedlot Probiotic LAB. Strains Compatibility

**FIGURE 1:**
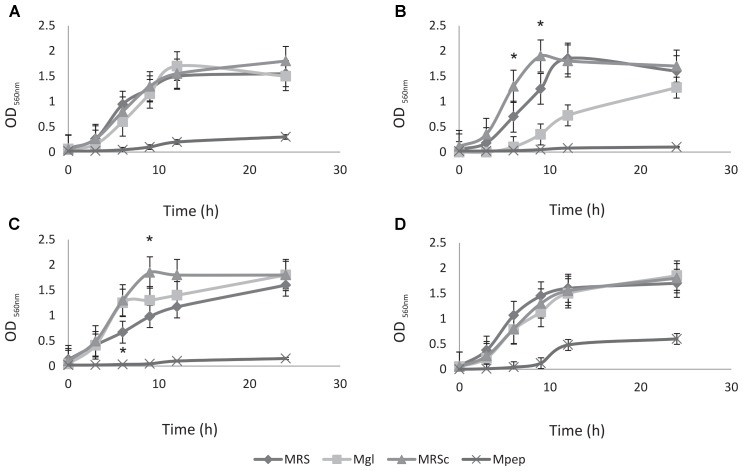
Growth kinetics of probiotic lactobacilli strains in different culture media. **(A)** *Lactobacillus acidophilus* CRL 2074, **(B)** *L. amylovorus* CRL 2116, **(C)** *L. mucosae* CRL 2069, and **(D)** *L. rhamnosus* CRL 2084.

Preliminary experiments to optimize the large-scale production required to deliver high numbers of probiotic live bacteria to feedlot cattle were performed; large-scale and low cost production of these bacteria is becoming an important issue. Therefore, the ability to produce a large number of cells, growth parameters and the use of low cost media ingredients should be considered for growth medium optimization. The selection of feedlot strains to be applied as probiotics previously carried out (Maldonado et al., 2018) together with safety traits (this study) allows the selection of *L. acidophilus* CRL2074, *L. amylovorus* CRL2116, *L. mucosae* CRL2069 and *L. rhamnosus* CRL2084 for a preliminary screening of optimal culture conditions to produce high cell mass. For this purpose, five different media involving several nitrogen (skim milk, soluble protein concentrate from whey, peptone casein, yeast extract) and carbon sources (glucose, lactose) were assayed, their composition being shown in **Table 2**. Results showed a high dependence of lactobacilli growth on the composition of the different evaluated culture media (**Figure 1** and **Table 5**). Different kinetics were displayed both measuring OD and CFU/mL counts. When OD_560_ max was determined in clear media (Mpep, Mgl, MRSc and MRS), *L. acidophilus*, *L. amylovorus* and *L. mucosae* were not able to grow in Mpep medium containing peptone, while *L. rhamnosus* exhibited a slight growth (**Figure 1**). A better growth was found for all four lactobacilli in Mgl medium (containing glucose) and higher OD_560_max were exhibited by *L. mucosae* and *L. rhamnosus* at 24 h. Nevertheless, maximal OD values were also observed for lactobacilli when MRSc was used; MRS (free pH) values were somewhat lower. When growth parameters were calculated from counts (CFU/mL) obtained by plate-dilution method, lactobacilli yielded the highest growth (>10^9^ CFU/mL) when inoculated in Mgl, Mlac, MRS, and MRSc media; lower growth was obtained in Mw and Msm media while mostly poor growth was produced in Mpep medium. Highest cell numbers were reached in Mgl (*L. acidophilus* and *L. mucosae*), Mlac (*L. acidophilus*, *L. amylovorus*, and *L. rhamnosus*) and MRS (*L. acidophilus* and *L. mucosae*). As reported by Manzoor et al. (2017), even when MRS medium represents a rich and suitable condition to support optimal lactobacilli growth, its high formulation cost and potential environmental hazards make it unviable for large-scale commercial applications. From our results, Mlac (g/l: skim milk, 10; yeast extract, 10; lactose, 30 pH: 6.5) and Mgl (g/l: yeast extract, 20; glucose, 10 pH: 5.9) showed the best conditions for the semi-industrial production of selected feedlot probiotic lactobacilli (**Table 5**). The presence of sodium acetate in Mgl medium, a component of commercial MRS medium, was reported as energy source and selective agent for lactobacilli (Stiles et al., 2002). On the other hand, whey protein concentrate (protein, 78%; carbohydrates, 4.5%) medium (Mw) as well as skim milk containing medium (Msm), a nitrogen (casein, ∼35%) and carbon (lactose, ∼50%) source respectively, were not able to produce high lactobacilli biomass. This result disagrees with economic and growth advantages of skim milk-based media used for LAB biomass production (Kusnadi and Afriyan, 2012). In coincidence to that found by measuring OD (**Figure 1**), the lowest biomass production by the selected lactobacilli was obtained in peptone casein containing medium (Mpep), *L. acidophilus, L. amylovorus*, and *L. rhamnosus* displaying the lower CFU/mL max values (**Table 5**). Particularly for *L. acidophilus*, Olson and Aryana (2012) reported a growth decrease in the presence of peptone compared with skim milk in coincidence with results from this study. Modified media composed by yeast extract, glucose and sodium acetate/sodium glutamate as major ingredients omitting peptone (expensive nitrogen source) were used for biomass production by fecal *L. plantarum* strains intended to be used as probiotic (Hwang et al., 2011). Optimized media containing agro-industrial residues such as cheese whey, industrial yeast extract, corn steep liquor, soybean meal and molasses among others, were assayed for lactobacilli biomass production (Hwang et al., 2011; Chiang et al., 2015; Manzoor et al., 2017). In view to be used as probiotic mixture, a final *Lactobacillus* strains compatibility was carried out. Results indicated that there was not inhibition of one strain on the growth of another.

**Table 5:** Growth parameters of selected probiotic lactobacilli strains.

Strains	Growth parameters	Culture media						
		Mpep	Mgl	Mlac	Mw	Msm	MRS	MRSc^†^
	μ (h^-1^)	0.06	0.76	0.67	0.10	0.13	0.48	0.31
*L. acidophilus* CRL2074	Growth potential	0.07	4.97	4.40	0.22	0.30	2.12	1.35
	CFU/ml max	1.20 × 10^3^	2.80 × 10^9^	2.80 × 10^9^	2.00 × 10^5^	1.00 × 10^5^	1.08 × 10^9^	1.20 × 10^8^
	OD_560__nm_ max	0.30	1.70	ND	ND	ND	1.50	1.80
	μ (h^-1^)	0.05	0.22	0.39	0.15	0.18	0.45	0.84
*L. amylovorus* CRL2116	Growth potential	0.11	3.13	4.11	1.60	2.14	1.97	3.67
	CFU/ml max	1.30 × 10^4^	9.55 × 10^7^	1.29 × 10^9^	4.00 × 10^6^	1.40 × 10^7^	5.14 × 10^8^	9.70 × 10^8^
	OD_560__nm_ max	0.10	1.30	ND	ND	ND	1.80	1.90
	μ (h^-1)^	0.08	1.10	0.36	0.78	0.10	0.51	0.44
*L. mucosae* CRL2069	Growth potential	0.18	4.78	2.39	5.14	1.00	1.29	0.96
	CFU/ml max	1.70 × 10^5^	6.03 × 10^9^	8.90 × 10^8^	1.40 × 10^8^	1.00 × 10^6^	1.62 × 10^9^	1.23 × 10^8^
	OD_560__nm_ max	0.15	1.80	ND	ND	ND	1.60	1.8
	μ (h^-1^)	0.20	0.58	0.56	0.42	0.36	0.62	0.38
*L. rhamnosus* CRL2084	Growth potential	2.08	0.75	2.44	1.86	1.57	1.11	1.69
	CFU/ml max	2.48 × 10^7^	3.24 × 10^8^	1.12 × 10^9^	1.80 × 10^8^	2.00 × 10^8^	7.55 × 10^8^	8.70 × 10^8^
	OD_560__nm_ max	0.60	1.85	ND	ND	ND	1.70	1.90

*ND, no determined; ^†^MRSc, MRS pH controlled; OD_560*nm*_ max was determined only in clear media at the incubation time indicated in brackets*.

## Conclusion

From this study, LAB isolated from steers feces, soil pens and feed rations were found as a reservoir of AR and virulence genes. However, *L. acidophilus* CRL2074, *L. amylovorus* CRL2116, *L. mucosae* CRL2069 and *L. rhamnosus* CRL2084 were able to be selected as probiotic candidates being free of AR and virulence factors, reaching high cell numbers in optimal culture media and compatible among them. These strains, alone or in combination, are being administered to feedlot steers for *in vivo* studies to elucidate their health and productivity benefits.

## Author Contributions

FM and NM performed the laboratory phenotypic antibiotic test. CA carried out molecular work for antibiotic and virulence genes investigation. HM was responsible for culture conditions optimization. MN-M and GV organized experimental protocols, analyzed the data, discussed and wrote the manuscript. All the authors reviewed the manuscript.

## Conflict of Interest Statement

The authors declare that the research was conducted in the absence of any commercial or financial relationships that could be construed as a potential conflict of interest.
